# Online public health promotion at the local level: An evaluation of four local authority-led marketing campaigns

**DOI:** 10.1177/20552076231220151

**Published:** 2024-01-03

**Authors:** Kristin Hanson, Anna-Marie Degas, Daniel Green, Antoine Al-Hosri, Tushna Vandrevala

**Affiliations:** 1 Department of Psychology, 145285 Kingston University, London, UK; 2 102116 Department of Health Behaviours and Public Health Services, Royal Borough of Kingston upon Thames, Kingston upon Thames, UK; 3 170925 Faculty of Health, Science, Social Care and Education, Kingston University, London, UK

**Keywords:** Public health, health promotion, social media marketing, search engine marketing, programmatic marketing, online advertising, behaviour change, local authority, COM-B, ethnicity

## Abstract

**Objective:**

Local authority-led online campaigns offer the possibility of targeted health promotion to connect local services and residents. This study assesses the evidence for medium (e.g., click-trhoughs) and high (off-line behaviour change) levels of public engagement with four local authority-led campaigns across a variety of public health promotions (sexual health, weight loss, and vaccination), online marketing approaches (social media marketing, search engine marketing, and programmatic marketing) and target demographics (language, gender, age, income, ethnicity) undertaken by a London borough local authority.

**Methods:**

Employing quasi-experimental and observational study designs, engagement with local health services during the course of the campaigns was evaluated. The first three campaigns were evaluated based on an interrupted time series model of intervention assessment comparing outcome variables of interest during the campaign to periods before and after the campaign period. The results of the fourth campaign, an observational case-study, are discussed using descriptive statistics only.

**Results:**

The analyses of the high engagement data for two of the three campaigns statistically assessed clearly supported the effectiveness of the campaigns. While the effect of high engagement could not be determined in the other two campaigns, they provide data that may be useful in online campaign design.

**Conclusions:**

The evidence assessed in this study across a variety of platforms, health promotion initiatives, and population targets suggests that local authority-led online marketing campaigns for health promotion may be useful for increasing participation in public health programmes.

The Health and Social Care Act of 2012^1^ gave local authorities in the United Kingdom (UK) new responsibilities and freedoms to improve and protect the health of their local populations. This move empowered local authorities to capitalise on their knowledge of the local population, allowing for the design of health promotion interventions that are tailored to specific segments of the population.^2^ This approach of place-based interventions led by local authorities brings primary preventative strategies in closer contact with the residents, potentially engaging people before disease onset.^3,4^ For example, engagement with a local fall prevention service may delay the need for social care, or attending a local smoking cessation event may prevent later related healthcare services. This early, preventative engagement is designed to maximise residents’ health literacy and agency and to decrease overall healthcare costs. Increasing local participation in public health programmes is therefore a key objective for local authorities. In an effort to narrow health inequality gaps, place-based approaches have emerged as an important foundation for producing population-level changes when supporting the most deprived areas with the poorest health.

The COM-B framework for developing and evaluating digital interventions and promoting behaviour change in health and social care^5,6^ is a widely employed tool used to assess and design interventions that aim to increase participation in public health programmes.^7^ The framework proposes a ‘behaviour system’ in which behaviour proceeds from three key components: Capability (C), Opportunity (O), and motivation (M). Capability refers to an individual's capacity (both psychological and physical) to engage with the behaviour. The motivation component expands on the ideas of conscious decision-making and goals to include all brain processes that direct and energise behaviour, which would include reflective (beliefs, intentions, and goals) and automatic (emotions, feelings, and habits) processes. The third component, opportunity, refers specifically to those environmental (time, location, and resources) and social (social norms and social cues) elements that lie outside the individual and that either prompt or make the behaviour possible.

In the case of participation in public health programmes, the facilitators and barriers to capability may include removing the psychological stigma of sexual health testing, or language barriers to information. Public health programmes for weight loss or smoking cessation may seek to capitalise on motivational factors such as saving money or improving one's mobility and energy levels. To address barriers to this opportunity component, localised health promotion initiatives to increase participation in public health programmes often centre not only on traditional advertising means such as pamphlets and posters, but also on embedding prevention into place-based systems and processes that have proven engagement. Strategies of health promotion often include those that rely on communication channels within the health system – such as referrals from health professionals – but may also utilise established communication systems within the community to reach residents who may be less likely to engage with the health system, such as promotion though health advocates in the community and religious centres.^8^ Locally led digital marketing may provide a unique instrument by which to address potential ‘location’ barriers to opportunity by increasing accessibility as well as by cueing an individual's current local social environment. Online health promotion initiated at the local authority level extends the reach of public health into established communication systems and may offer an economical tool to reach residents who may not otherwise engage with other modalities of health promotion.

In the UK, at least 60% of the population uses the internet to access health information.^9^ Online platforms may also afford new possibilities for engaging members of the public for whom in-person or even telephonic inquiries and consultations carry with them the fear of stigmatisation, discrimination, or fears surrounding immigration status. This may be particularly important for people who seek to access information relating to mental and sexual health issues, which carry a degree of stigma in some communities.^10–13^ Online health promotion can also extend the reach of messages through the ability of the internet to overcome physical barriers.^14^ In addition, this mode of health promotion allows for messages to be targeted and to be used to combat disinformation through timely contact. In spite of its advantages, residents’ engagement with local authority-led online health promotion initiatives is less explored and evidence of behaviour change is scarce. In the current study, we evaluate four online, local authority-led health promotion campaigns – including social media marketing, search engine marketing and programmatic advertising campaigns – and trace these to their behaviour outcomes. The four campaigns evaluated were Come Correct (safer sex), Second Nature (weight loss), Sexual health testing (at-home testing kits), and COVID-19 vaccination.

## Measuring the impact of online marketing in health promotion

Whilst the advantages of digital marketing seem intuitive, measuring this new ability to reach the population is less straightforward.^15^ User impact measures captured by online platforms often include the number of times the advertisement has been seen and interacted with by users. These measures are discussed in terms of the number of *impressions* (the number of times an advertisement has been seen), *reach* (the number of individual users who have seen an advertisement at least once), *likes* (a user's indication of agreement with a posting), *shares* (a user's intentional further dissemination of the posting) and *click-throughs/swipe-ups* (the number of user interactions that activate a link with another website, e.g. a public health website). In an attempt to capture behaviours that can be causally linked to particular health promotion social media postings, key performance indicators have been proposed.^16,17^ Mere acknowledgement of content (e.g. likes) is considered a *low engagement* activity; actively engaging with the posting (e.g. shares, click-throughs, swipe-ups) is considered to be *medium engagement*; and *high engagement* relates to actual participation in off-line interventions as a result of exposure to a social media posting (see Table 1).

**Table 1.** Online marketing measures and related levels of engagement*.*

Level of engagement	Definition	Metric
Low	Indications of agreement with a posting	Likes
Medium	Interactions with an advertisement	Clicks/swipe-ups
High	Adoption of a promoted behaviour	Offline engagement

In spite of the theoretically significant potential for digital marketing in health promotion, evidence of its effectiveness is often limited to low and medium engagement metrics. These likes and shares are acknowledged as inadequate measures of behavioural change: one might ‘like’ or ‘share’ a post for a multitude of reasons including affection for the source of the message, or even the message's accompanying photo. On the other hand, individuals who haven’t engaged with the post may be impacted by the message, referred to by Steffens et al.^18^ as the ‘silent audience’. Medium levels of engagement provide stronger evidence but are still removed from behaviour. These limitations of measurement for health promotion campaigns mean that social media are ‘often characterised as having potential rather than proven to be effective’.^19^

While the literature supports the impact of campaigns on these low and medium engagement metrics, evidence that digital campaigns contribute to high levels of engagement (i.e. behavioural change or adoption of behaviour) is less common. For example, a review of social media interventions related to sexual health found that evidence of impact was largely limited to low measures of engagement^20^ and estimates of behaviour change (high levels of engagement) were commonly limited to the verification of impact in participants’ self-reports of, for example, condom use after exposure to a Facebook page campaign.^21^ Similarly, a scoping review of social media interventions that sought to increase cancer screening^17^ found that only one of the 23 studies measured health behaviour beyond intention and knowledge, without addressing changes in behaviour.

Access to information on health promotion campaigns and the uptake of health services means that local authorities are well placed to contribute to the evidence surrounding the effectiveness of health promotion interventions. A study that employed a Facebook advertisement to promote home-based chlamydia testing found a 277% increase in click-throughs to the testing kit page (medium engagement) and a 41% increase in test kit orders (high engagement).^22^ Likewise, a Breast Cancer Screening Facebook page run by a Health Improvement Practitioner, was found to be associated with a 12.9% increase in the number of women attending their first screening between screening cycles in Stoke-on-Trent.^23^ Whilst this evidence is encouraging, it is limited. The purpose of the current study is to expand on the evidence base to demonstrate the scope of possibilities available to local authorities by evaluating local authority-led online campaigns with metrics of high engagement where available. The public health online campaigns run across a number of initiatives (sexual health, weight management, and vaccination), targeted populations (age, geographic, gender, and ethnicity) and advertising platforms (social media, websites, and search engines).

## Social media marketing, search engine marketing, and programmatic advertising for health promotion

In this study, we evaluate four online health promotion initiatives that span social media marketing, search engine marketing, and programmatic online advertising, undertaken by the Royal Borough of Kingston-upon-Thames (Kingston Borough) between 2020 and 2022. Kingston Borough has a population of 176,107, and approximately one-third of Kingston Borough residents are from Black, Asian, and minority ethnic backgrounds.^24^

### Social media marketing

The use of social media websites and applications such as Instagram, TikTok, X (formally known as Twitter), and Facebook has become an integral part of day-to-day communication. In the UK, 84.4% of people access social media every day.^25^ This level of public engagement with social media offers an opportunity to reach a large number of health information users, including those who may not fully engage with other public health modalities, and to target the recipients of health promotion messages more accurately.

Social media marketing allows for a level of population segmentation that was not available before the widespread use of these platforms. Like commercial organisations, public health authorities can now use social media to direct campaign messages to populations with particular demographic characteristics (ethnicity, gender, age, and geographic location). For example, it is possible to target all men aged 18+ in the most deprived postcodes in the borough. This targeting is especially valuable because health promotion messaging can be culturally bound;^26^ messages may therefore be tailored to a particular language or culture, aiming to reach those who may engage less with mainstream sources of health information.

While the reach and targeting available through social media allow public health organisations to enhance the effectiveness of health promotion interventions for all populations, the potential utility of social media marketing to underserved populations – such as minoritized groups and young people – may be particularly potent as the social media platform preferences in these groups vary from other populations.^27,28^ In addition, the provision of accurate and timely health information on these platforms is critical to combat health misinformation that tends to be more prevalent on social media sites than on other health information sources.^29^

### Search engine marketing

The selection and ranking of websites by search engines play a vital role in online health information-seeking behaviour.^30^ Search engine marketing – in which entities pay for a particular link to appear prominently in the search engine's results when particular words or phrases are searched for – provides a potentially powerful means of promoting a particular health message. It is also a useful approach to combat misinformation as the ranking of search engine results plays a significant role in online health information-seeking behaviours and attitudes, even independent of users’ evaluation of the source credibility.^30^ This public health marketing strategy targets those users specifically interested in a particular health topic and can even be set to provide translated advertisements in the language in which a user's browser is set. For example, by including messages translated into Polish as part of a campaign, it is possible to target users who have set their browser settings to Polish also. By providing translated messages as part of a campaign, a significant barrier to health literacy is removed.^31,32^

### Programmatic online advertising

Programmatic advertising is the automated purchase of advertising which allows impressions to be split across multiple platforms to target a segment of the population. Typically purchased through an agency, advertising space is purchased based on the most economical method of reaching a particular target audience through automatic bidding auctions. The advertisement impressions (the number of times an advertisement is viewed) may include appearances on social media, on search engine search results, or on targeted websites. This wide allocation, to include targeted websites, is particularly useful when wanting to reach immigrant populations: users may have their browsers set to the English language but may follow news or sports information from their home country. Programmatic advertising allows for the allocation of impressions to include advertisements on websites that would be relevant to different cultural and language groups.

## The current study

The potential for locally led online campaigns to affect increased participation in public health initiatives is significant, but the evidence of this potential has been more difficult to establish. Employing a combination of quasi-experimental and observational designs, we assess four online public health campaign case studies undertaken by a London borough local authority – Come Correct, Second Nature, Sexual Health Testing, and COVID Vaccination – across a range of platforms, targeted behaviours, and populations. While Campaigns 1–3 demonstrate the impact of online campaigns on behaviour, Campaign 4 provides a case study of the breadth of online marketing campaigns available to local authorities, though without the benefit of high engagement metrics. By evaluating the relationships between these campaigns and increased participation in public health programmes, this study seeks to contribute to the evidence base for locally led online marketing as a useful tool for increasing the public's opportunity for behaviour change.

## Method

The Public Health team at Royal Borough of Kingston-upon-Thames (Kingston Borough) undertook four discrete online marketing campaigns to assess the impact of locally led online public health interventions on participation in public health programmes. The campaigns were undertaken over a period from July 2020 through April 2022. The first campaign, Come Correct, was a social media marketing campaign for a service that provides free condoms and sexual health advice to young people in the borough. A second social media marketing campaign, Second Nature, targeted men in the borough and promoted a weight management programme. The third campaign was a search engine marketing campaign aimed at encouraging residents to access sexual health testing online, and a fourth campaign employed programmatic advertising to promote the COVID-19 vaccination. Table 2 outlines each of these campaigns.

**Table 2.** Overview of public health campaigns*.*

	Come Correct Condom Scheme	Second Nature	Sexual Health	COVID vaccination
Public health initiative	Safe sex	Weight loss	Sexual health testing	COVID vaccination uptake
Marketing type	Social media	Social media	Search engine	Programmatic advertising
Platforms	Instagram and Snapchat	Instagram and Facebook	Google	Facebook, Instagram, Snapchat, Google, targeted websites
Target population	16- to 24-year-olds	Men	All adults	Ethnic minorities and English as an additional language
Campaign dates	15–21 July 2020	25 May–7 November 2021	7 January–31 March 2022	December 2021–February 2022 April 2022
Engagement metric	33 new service registrations	68 new service referrals	2262 testing kits ordered	6503 visits to the NHS booking website
Level of engagement	High	High	High	Medium
Cost	£140	£385.34	£458	Approx. £1400–£1700

Data collection for each of the campaigns drew upon anonymous, aggregated data that was subject to the information-sharing terms and conditions of the providers. Obtaining consent specifically for the purpose of this study was therefore unnecessary. We evaluated the first three campaigns based on an interrupted time series model of intervention assessment,^33^ comparing outcome variables of interest during the campaign to periods before and after the campaign period. Where inferential statistics were possible, we used generalised linear models with negative binomial regressions to assess the statistical significance of the period differences. High engagement measures were not available for the COVID Vaccine campaign and therefore did not lend itself to this type of analysis. The campaign is included as a case study to illustrate the additional tools of translation and programmatic advertising to remove barriers to opportunity. The results of this campaign are discussed using descriptive statistics only. All analyses were conducted using jamovi 2.3.^34^

### Come Correct Condom Scheme

#### Design and intervention

The London-wide Come Correct programme is a safer sex campaign that provides access to free condoms and sexual health advice to registered young people aged 13 to 24. A young person must live or attend education in one of the participating London boroughs in order to be able to register. Upon registration, young people are provided with a card (known as a C-Card) which can be presented at pharmacies and other participating outlets displaying the Come Correct logo to receive free condoms and sexual health advice.

To promote the Come Correct service, Kingston Borough undertook an Instagram and Snapchat advertising campaign from 15 to 21 July 2020. The campaign targeted platform users in the 13 to 24-year-old age group, identified as local to the borough. The advertisements, which comprised the simple text ‘Free Condoms – Quick and Easy Signup’ (Instagram) or ‘Free Condoms – Swipe up to Sign up’ (Snapchat), directed those who swiped or clicked to a Kingston Borough-specific page on the Come Correct C-Card registration website. Illustrations of the advertisements are included in Appendix A. Advertisements cost £70 for each platform (a total of £140) and were displayed on Instagram (both feeds and stories) and on Snapchat.

#### Data collection and analysis

The link provided in the adverts contained a unique tracking module (UTM) so that traffic to the C-Card website and new service registrations (high engagement) generated by the interactive adverts could be accurately attributed to the Kingston borough campaign. Data for reach (low engagement) and link clicks (medium engagement) for the period of the campaign was collected from Facebook (for Instagram) and Snapchat analytics queries.

This quasi-experimental study evaluated the impact of the campaign on registrations for the C-Card using an ABA-interrupted time series analysis of data comparing two weeks before and after the campaign to the campaign period itself. In light of the low behavioural commitment required by this campaign, we hypothesised a significant level change in the number of registrations during the campaign period as compared to the periods before and after.

Due to the lack of registrations in the comparative periods of two weeks before and after the campaign (indeed, there were a total of two new C-Card registrations during the two months preceding the campaign), the data did not lend itself to inferential statistical analysis. The campaign was therefore assessed using descriptive statistics.

### Results

In total, the Instagram advertisements reached 7961 and the Snapchat advertisements reached 7039 people, of which 240 interacted with the Instagram advertisement (3.0%) and 469 with the Snapchat advertisement (6.7%) over the period of the 5-day intervention. The advertisements resulted in 709 visits to the C-Card registration website (see Table 3). Of those who interacted with the Instagram advertisement, 45% were between 13 and 17 years of age, while 78% of those who interacted with the Snapchat advertisement were in this age group.

**Table 3.** Come correct campaign metrics.

Platform	Click-throughs/swipe-ups	Reach
Instagram	240	7961
Snapchat	469	7039
Total	**709**	**15,000**

**Figure 1.**
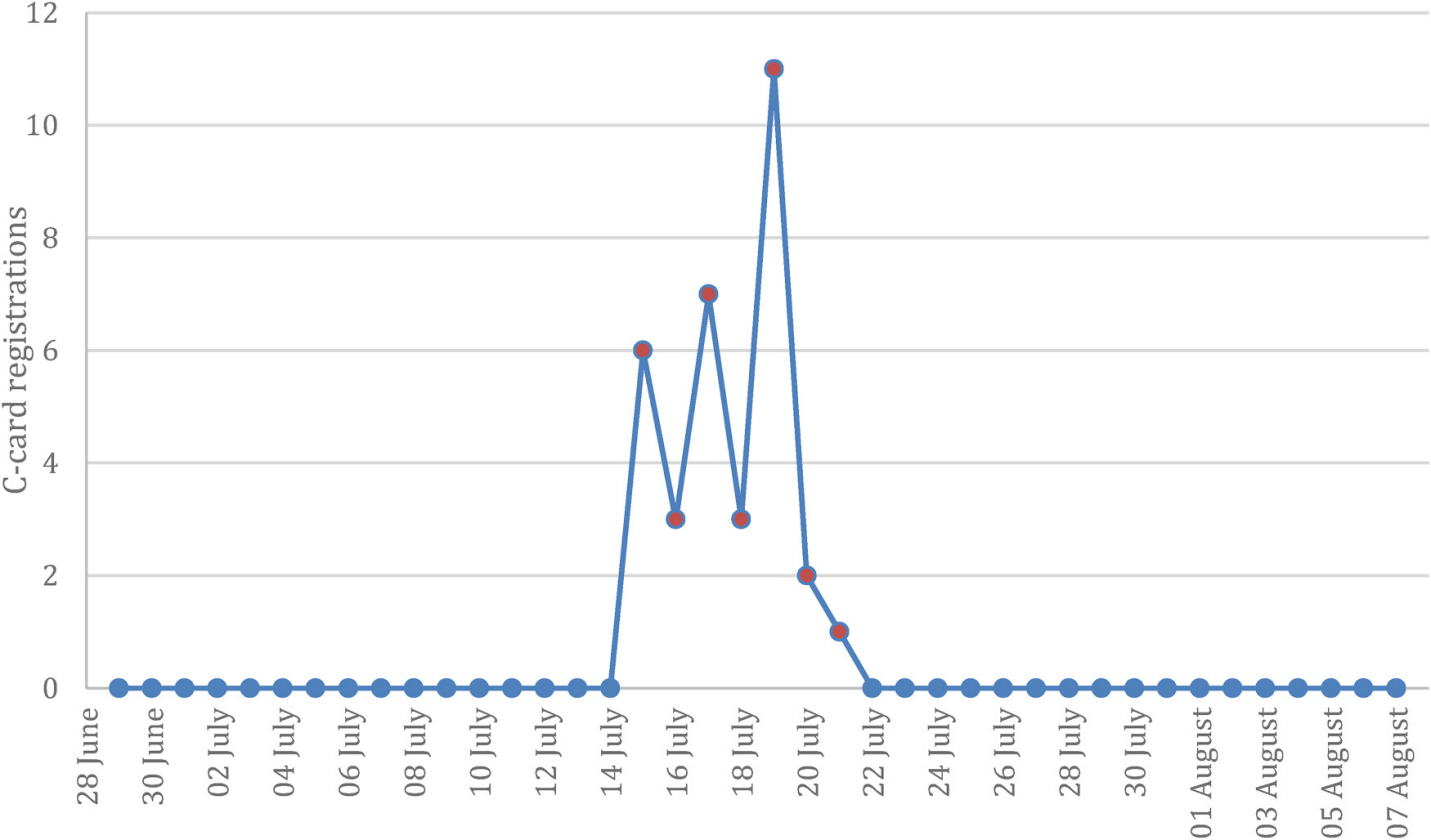
C-Card registrations including two weeks before and two weeks after campaign (15 July–21 July 2020). Active campaign period observations indicated in orange.

There was a total of 33 new C-Card registrations during the one-week campaign period as compared to no new registrations in the two weeks before and after the campaign. Figure 1 visually depicts this contrast. The cost of the advertisements per registration in the period was £4.24.

### Campaign 2: Second Nature

In contrast to the C-card campaign, where the user is only registering for the ability to receive condoms, Second Nature registration represents a commitment to a year-long weight loss programme (a more long-term and complex commitment).

#### Design and intervention

Second Nature is a national behavioural science-based weight management programme officially backed by the UK's National Health Service. The digitally supported weight loss programme is purposely constructed to not appear to primarily target women and is therefore seen as an alternative to other national weight loss services. The campaign run by Kingston Borough was undertaken upon the borough's adoption of the local service for the programme to encourage men in the borough to register for the service.

The Facebook and Instagram campaign utilised advertisements with the text ‘Welcome to Second Nature – an intelligent way to improve your habits and your health’ and ran for a month, from 25 May to 24 June 2021, with two further booster campaigns which ran from 15 July to 15 August and from 7 October to 7 November 2021. Illustrations of the advertisements can be found in Appendix B. The advertisement agreement prescribed a targeted audience of males aged 18+ located in Kingston. People who clicked on the advertisements were automatically connected to a Kingston Borough-specific sign-up form for the service.

#### Data collection and analysis

The link provided in the advertisements contained a UTM so that traffic to the Second Nature website and new service registrations (high engagement) generated by the interactive adverts could be accurately attributed to the Kingston Borough campaign. Data for reach (low engagement) and link clicks (medium engagement) were obtained from Facebook and Instagram analytics. New service registrations (high engagement) for the period of the campaign were available to Kingston Borough through their designated sign-up page.

This quasi-experimental study evaluated the impact of the campaign on new registrations using an ABA-interrupted time series analysis of data. We used a generalised linear model with negative binomial regression to compare active and non-active campaign periods. A negative binomial model is similar to a Poisson model but includes an additional term to account for the excess variance in our data. Our proposed model hypothesised a significant increase in the number of new registrations during the active campaign period.

#### Results

In total, the advertisements reached 3708 women and 34,698 men, of which 64 (1.7%) of women and 958 (2.8%) of men interacted with the advertisement over the period of the intervention. The advertisement resulted in 1023 visits to the Second Nature registration page and a total of 68 new registrations between the first discrete advertising campaign in May 2021 and the final booster campaign in October 2021. The advertisements cost a total of £385.84 for the three advertising periods resulting in an advertising cost per new registration during the period of £5.67.

The daily average number of registrations during the active campaign period was 0.37 [95% CI: 0.21–0.53] (*SD* = 0.67) could not be distinguished from the daily average number of registrations during the period in which the campaign was not active (*M* = 0.28 [95% CI: 0.17–0.39], *SD* = 0.67). As indicated in Table 4, negative binomial generalised linear regression also indicated that the data could not provide evidence of a distinction between the non-active (A) and active (B) campaign periods.

**Table 4.** Regression of registrations completed on non-active (A) and active (B) campaign segments.

Effect	Estimate	SE	exp(B)	95% Exp(B) CI	z	*p*
(Intercept)	−1.13	0.15	0.32	[0.24–0.43]	−7.38	<.001
B–A	0.27	0.31	1.31	[0.72–2.40]	0.88	0.38

**Figure 2.**
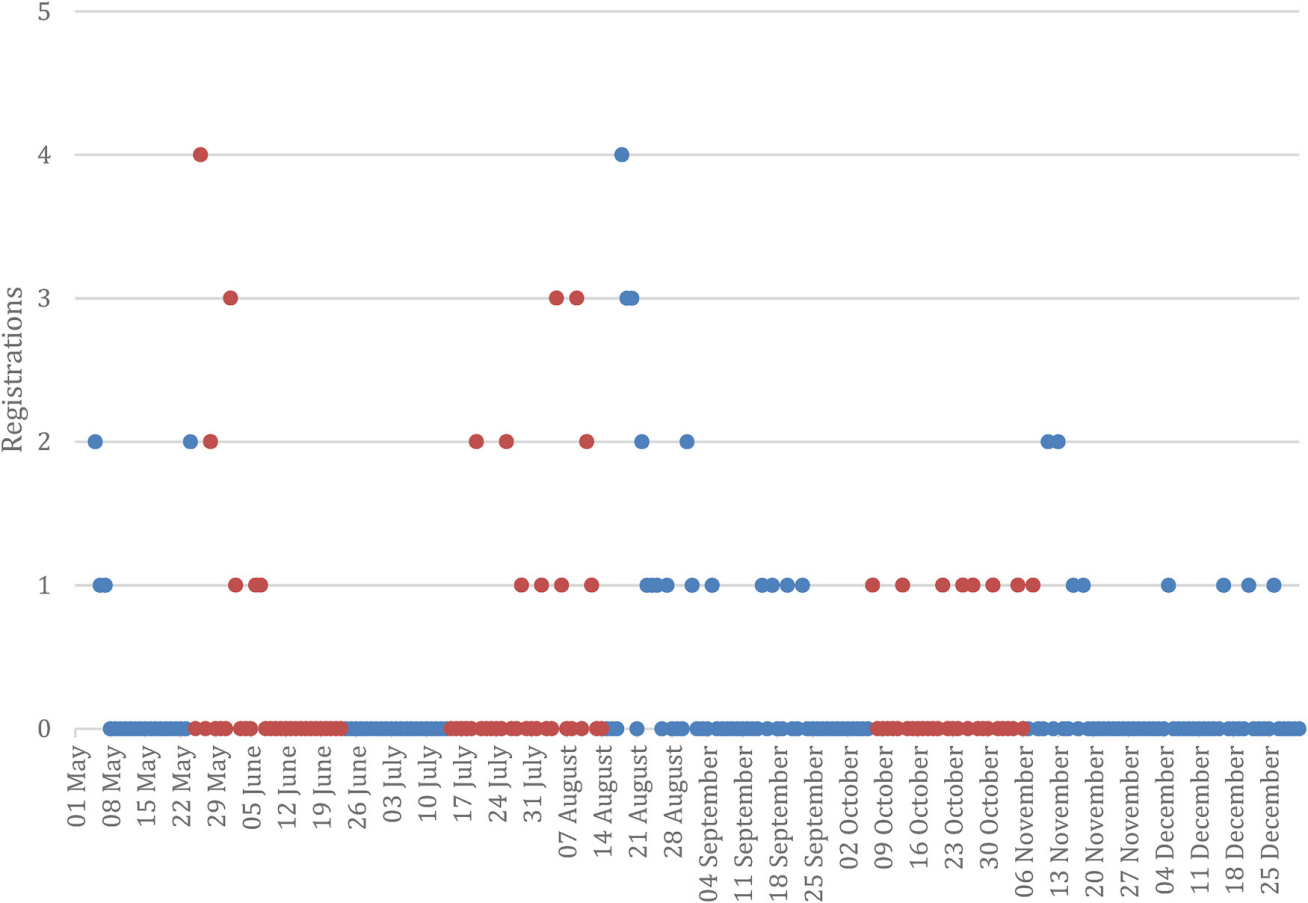
Second Nature registrations from May through December 2021. Active campaign period data indicated in orange.

The regression indicates that registrations were 31% more likely during the active campaign period than in the non-active campaign period. But the 95% CI ranges from 28% less likely to 1.40% more likely to register, and this predictor did not meet the threshold of significance (*p*  = .38). We can therefore not draw any strong statistical inferences from this data, and the hypothesis is therefore not supported. The requested behaviour for the Second Nature campaign may have had an effect on the delineation between the registrations in the active and non-active campaign periods, however. The year-long programme commitment may have resulted in more deliberation by the user, resulting in registrations just after the active campaign period: 12 of the 68 registrations were in the week following the first booster campaign, and another five in the week after the second booster campaign (refer Figure 2), accounting for 25% of the registrations over the seven-month period. The inclusion of an additional week for these two campaign periods–assessed on an exploratory basis, and only for illustration–indicates a significant effect of the campaign in which there was 24% ([95% CI: 3% - 48%], *p *= .02) greater likihood of registration during these periods. 

### Campaign 3: Sexual Health

This campaign sought to increase the uptake of online testing to relieve some pressure on borough sexual health clinics by reducing in-person attendance for routine testing. The aim was to divert patients away from booking face-to-face appointments and toward ordering sexual sexually transmitted infection kits online. For this search engine marketing campaign, Kingston Borough paid for an advertisement to appear at the top of Google search results when local users aged 18+ searched for sexual health advice online. The advertisement linked to the website Sexual Health London, an online testing service from whom home testing kits could be ordered in 30 Boroughs across London. This campaign ran from 7 January to 31 March 2022 and provided users with a Kingston Borough-specific link to the website. The cost of the advertisement for the period was £458.

#### Data collection and analysis

Data for reach (low engagement) and link clicks (medium engagement) for the period of the campaign was collected from Google Analytics. Unlike the first two campaigns, data for service registration (high engagement), online kits ordered (offline behaviour change) and used testing kits returned during the campaign was provided by means of a custom URL provided by Sexual Health London embedded in the interactive advertisements. Users who clicked the link in the advert were directed to an exclusive part of the Sexual Health website, which Kingston Borough could access, to ensure that figures for kits ordered via the campaign alone could be accurately collated. This eliminated the possibility of including any service registrations and orders from outside of the interactive advert campaign.

This quasi-experimental study evaluated the impact of the campaign on new users, kits ordered, and tests completed using an ABA-interrupted time series analysis of data. We used a generalised linear model with negative binomial regression to compare pre-, active, and post-campaign periods. Our proposed model hypothesised a significant level change in the number of new users, kits ordered, and tests completed during the active campaign period.

#### Results

In total, the advertisements had a total of 31,362 impressions (17,807 women and 9010 men), of which 23% interacted with the advertisement by clicking through to the site over the period of the intervention. The advertisement resulted in 7155 visits to the testing website (4150 women, 2116 men, and 889 unknown) and a total of 3754 new testing kit orders (52% of click-throughs) in the months of the campaign. The cost of the advertisement per testing kit ordered over the months of the intervention period was £0.12. Of the testing kits ordered, 2843 sexual health tests were completed (76%).

As indicated in Table 5, new users, kits ordered, and tests completed all had higher averages during the campaign period than in the non-campaign periods. Average new users during the campaign (*M* = 96.4, *SD* = 19.7), were higher than in the pre-campaign period (*M* = 51.5, *SD* = 15.3) and in the post-campaign period (*M* = 64.4, *SD* = 21.5). Similarly, the average number of kits requested during the campaign (*M* = 96.4, *SD* = 19.7), were higher than in the pre-campaign period (*M* = 51.5, *SD* = 15.3) and in the post-campaign period (*M* = 64.4, *SD* = 21.5). And finally, the average number of tests completed during the campaign (*M* = 230, *SD* = 56.5), were higher than in the pre-campaign period (*M* = 96.8, *SD* = 25.7) and in the post-campaign period (*M* = 142, *SD* = 36.1).

**Table 5.** Descriptive statistics for new users, kits ordered, and tests completed by the campaign phase.

	Campaign	New users	Kits ordered	Tests completed
Mean	Pre (A)	51.5	124	96.8
	Active (B)	96.4	306	230
	Post (C)	64.4	160	142
Standard deviation	Pre (A)	15.3	37.6	25.7
	Active (B)	19.7	73.4	56.5
	Post (C)	21.5	53.1	36.1

*Note.* Pre (A) = the two months preceding the campaign (November and December 2021), Active (B) = the campaign period (January through March 2022), Post (C) = the two months following the campaign period (April and May 2022).

Our negative binomial generalised linear model of regression demonstrated a significantly higher number of new users, kits ordered, and tests completed during the active campaign period (B) as compared to both the pre-campaign period (A) and the post-campaign period (C). Refer Table 6.

**Table 6.** Regression of new users, kits ordered, and tests completed on campaign segments.

Outcome	Campaign segment	Estimate	SE	exp(B)	95% Exp(B) CI	z	p
New users	(Intercept)	4.21	0.05	67.15	[60.85 −74.24]	82.92	<.001
	A–B	−0.55	0.12	0.58	[0.45–0.73]	−4.49	<.001
	B–C	0.53	0.12	1.71	[1.34–2.17]	4.37	<.001
Kits ordered	(Intercept)	5.19	0.06	178.77	[160.43–199.91]	92.42	<.001
	A−B	−0.83	0.14	0.43	[0.33–0.57]	−6.13	<.001
	B−C	0.77	0.14	2.17	[1.66–2.83]	5.70	<.001
Tests completed	(Intercept)	4.96	0.05	142.50	[128.99–157.87]	96.27	<.001
	A−B	−0.82	0.13	0.44	[0.35–0.56]	−6.56	<.001
	B−C	0.62	0.12	1.85	[1.45–2.36]	4.96	<.001

*Note.* A: the two months preceding the campaign (November and December 2021); B: the campaign period (January through March 2022); C: the two months following the campaign period (April and May 2022).

For each of the outcome variables, the difference between the active campaign period and the not-active periods was a significant predictor (*p* < .001 for all outcomes). During the pre-campaign period, new users were 42% [95% CI: 27%–55%] less likely to register, and new users were 71% [95% CI: 34%–117%] more likely to register in the campaign period than in the post-campaign period. Similarly, during the pre-campaign period, kits were 57% [95% CI: 43%–67%] less likely to be ordered, and kits were 117% [95% CI: 66%–183%] more likely to be ordered during the campaign period than in the post-campaign period. Finally, during the pre-campaign period tests were 56% [95% CI: 44%–65%] less likely to be completed than during the campaign period, and tests were 85% [95% CI: 45%–136%] more likely to be completed during the campaign period than in the post-campaign period.

### Campaign 4: COVID-19 Vaccine campaign for higher-risk communities

#### Design and intervention

In December 2021, the UK government launched a COVID-19 vaccine to the public for the first time. To coincide with this, a national advertising campaign began to encourage the uptake of the vaccine among the population. With a large minority of Kingston Borough residents coming from Black, Asian, and minority ethnic backgrounds, Kingston Borough's public health team were conscious that the national advertisements might not reach this portion of residents as well as other high-risk populations. Therefore, to bolster the national campaign locally, Kingston Borough ran a series of advertisements to promote the COVID-19 vaccine to these groups and to attempt to combat vaccine-related disinformation.

To reach the target population efficiently, Kingston Borough partnered with an external agency to purchase programmatic advertising. The advertisements’ messages targeted ethnic minorities, pregnant women, and low-income residents. The programmatic advertising placed the advertisements across social media platforms including Facebook, Facebook Messenger, Instagram, and Snapchat. Search engine marketing was deployed as sponsored Google advertisements, where advertisements were placed at the top of relevant searches, such as ‘COVID vaccination’, or ‘vaccine side effects’. Within this advertising agreement, space was also purchased on websites that were likely to appeal to the diaspora in Kingston Borough including such sites as INT Sri Lanka, Pakistan News, News24 Albania, and TV Ghana. The campaign employed both English and non-English advertisements. English advertisements were translated into the top 11 languages that are requested regularly in the borough for translation services (Albanian, Arabic, Bulgarian, Farsi, Korean, Polish, Portuguese, Spanish, Tamil, Turkish and Urdu). The language of the advertisement (direct translations of the English language advertisements using the same imagery) that appeared to users was set to match their browser and app language settings. Both types of advertisements included a link that led users directly to the NHS vaccination booking website. This campaign ran from December 2021 until February 2022 and again in April 2022, and the programmatic advertising cost approximately £1400 to £1700.

#### Data collection and analysis

Data for reach (low engagement) and link clicks (medium engagement) for the period of the campaign was collected via Facebook, Snapchat, and Google Analytics. Kingston Borough was able to track clicks through to the NHS COVID vaccine booking website (medium engagement) via unique links. This campaign represents a case study demonstrating an alternative type of online campaign approach that may be useful in the design of interventions aimed at increasing representative participation in public health programmes. The data analysis was limited to verifying the data reported herein.

#### Results

**Figure 3.**
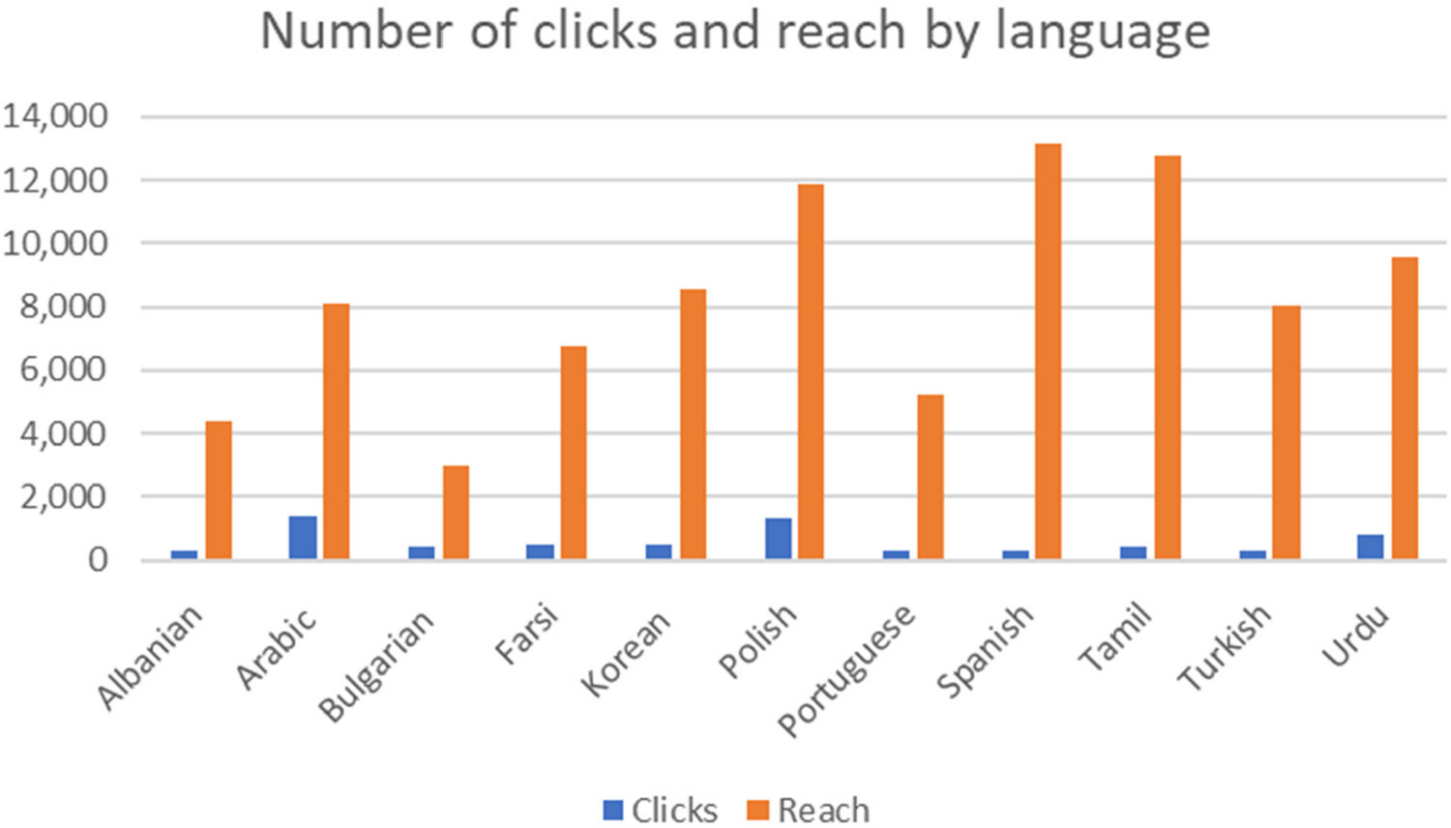
COVID-19 vaccine campaign metrics.

In total, there were 1,800,058 impressions and 14,077 click-throughs related to the programmatic advertising. Within this, metrics for certain targeted users were as follows. For language-specific targets, there were 700,801 impressions, the advertisements reached 91,344 people and 6503 (7.1%) of these clicked through to the NHS vaccine-booking website. Figure 3 presents the click-throughs and total reach for this element of the campaign by language.

For low-income users, there were 201,906 impressions, the advertisements reached 22,586 people, and 1261 clicked through to the NHS vaccine booking website. For pregnancy-relevant users, there were 30,484 impressions, the advertisements reached 4037 people, and 303 clicked through to the NHS vaccine booking website.

## Discussion

The various evidence assessed in this study suggests that local authority-led campaigns of online marketing for health promotion across a variety of platforms (e.g., social media, websites and search engines), health promotion initiatives (e.g., weight loss, sexual health, vaccines), and populations targets (e.g., young people, men, ethnic communities) may be useful in efforts to increase participation in public health programmes by offering increased opportunity for behaviour change through established communication systems. With insight into high-level engagement, this study provides evidence that these campaigns don’t just reach residents, but that they increase the desired behaviours.

By integrating into residents’ daily routines and removing barriers to opportunity, online interventions through social media, search engines, and website advertising may be able to achieve additional penetration into the local population and reach particular segments of the population that might previously not have engaged in health interventions. This integration represents an extension of place-based approaches to public healthcare by meeting residents where they are, therefore supporting an environment that increases access to healthcare. The findings related to the medium of communication are in keeping with the proposed impact of place-based health promotion^3^ and the impact of eliminating barriers to opportunity (O) proposed by the COM-B model of behaviour change.^5^ Future work may further explore the applicability of this model by utilising the theory's capability (C) and motivation (M) elements to design the content of online digital interventions.^6^

However, the findings in this study were limited not only by the quasi-experimental and observational designs that allowed only limited control of the structure of the campaign and non-campaign periods employed, but also by the rarity of the outcome data in the first two campaigns. While the descriptive statistics provided for the case studies are detailed and useful, such limitations impact the strength of the conclusions that can be drawn. To establish causality, future research may wish to undertake more tightly controlled experimental procedures to establish causality.

The Come Correct and Second Nature campaign results also demonstrated a contrast in the clarity of the effect of social media marketing for low- and high-commitment programme registrations. Future work may utilise the capability and motivational elements of COM-B to provide additional insight into understanding the time-to-decision differences for different programme characteristics such as these. Insights gained from such work may help refine online campaigns, analysis, and perhaps even the programmes themselves.

The use of online health promotion campaigns is highly scalable, and the campaigns employed by Kingston Borough are easily transferable to other local authorities and to the voluntary and community sector. Indeed, economies of scale could be achieved through cooperative advertisement development and coordinated campaigns. There are also opportunities that can be quickly capitalised upon by national health authorities, particularly in the area of search engine marketing. The mere awareness of services to members of the public may contribute to future uptake of services and recommendations, including prevention services, health-protective behaviours, and shifting to more economical services (e.g., at-home testing kits), creating both better health outcomes and economic efficiencies.

The potential for these types of health promotion campaigns at the local level has not been fully exploited. In the first instance, the advertisements in the COVID Vaccination campaigns were direct translations of English-language advertisements and were not specifically tailored for a local or culturally specific audience. Marketing research has suggested that ethnic and geographic identification cues can be employed to increase engagement with a campaign.^35,36^ Future A/B testing of tailored and non-tailored advertisements may provide insight into the most optimal messaging for particular groups. Building on this initiative, the co-design of digital marketing interventions – in which local residents are integral to the design of the health promotion campaigns targeted at their community groups – should be explored.

While the campaigns discussed here have demonstrated engagement with the population through text advertisements on a number of platforms, additional opportunities may be indentified by exploring other platforms and advertisement types. The reach and engagement with health promotion messages can be boosted through the use of memes and influencers,^37^ or through messaging types that may allow greater access on certain platforms including TikTok (whose audience is concentrated amongst young adults) and WhatsApp (the top reaching smartphone app).^28^ Future research may also wish to more precisely assess the degree to which these users are an audience shift or a new audience; in other words, the extent to which digital marketing allows local authorities to relieve the burden on other sources of health information and services and/or is reaching new/additional members of their population.

We have provided the costs per high-engagement behaviours including registrations with each of the relevant campaigns. Whilst the assessment of costs at this level provides important information, a more thorough cost analysis would require that information even further along the engagement journey is necessary. For example, it may be possible to identify levels of engagement with the Second Nature programme beyond initial registration, including weight loss and health statistics.

## Conclusion

While the evidence provided in this study cannot clearly establish causality due to its non-experimental nature, the analyses provided suggest that online marketing may positively impact participation in public health programmes. With refinement and expansion, digital marketing by local authorities holds a great deal of possibility and promise for a more inclusive public health service.

## Appendix A

Come Correct Advertisements

## Appendix B

Second Nature Advertisements
